# Carnauba wax nanoparticles enhance strong systemic and mucosal cellular and humoral immune responses to HIV-gp140 antigen

**DOI:** 10.1016/j.vaccine.2010.11.084

**Published:** 2011-02-01

**Authors:** Mauricio A. Arias, Andrew Loxley, Christy Eatmon, Griet Van Roey, David Fairhurst, Mark Mitchnick, Philip Dash, Tom Cole, Frank Wegmann, Quentin Sattentau, Robin Shattock

**Affiliations:** a Division of Clinical Sciences, St. George's University of London, London SW17 0RE, UK; b Particle Sciences, Inc., 3894 Courtney Street, Suite 180, Bethlehem, Pennsylvania 18017, USA; c The Sir William Dunn School of Pathology, University of Oxford, South Park Road, Oxford OX1 3RE, UK

**Keywords:** HIV, gp140 antigen, Nanoparticles, Mucosal immunity, Vaccines

## Abstract

Induction of humoral responses to HIV at mucosal compartments without inflammation is important for vaccine design. We developed charged wax nanoparticles that efficiently adsorb protein antigens and are internalized by DC in the absence of inflammation. HIV-gp140-adsorbed nanoparticles induced stronger in vitro T-cell proliferation responses than antigen alone. Such responses were greatly enhanced when antigen was co-adsorbed with TLR ligands. Immunogenicity studies in mice showed that intradermal vaccination with HIV-gp140 antigen-adsorbed nanoparticles induced high levels of specific IgG. Importantly, intranasal immunization with HIV-gp140-adsorbed nanoparticles greatly enhanced serum and vaginal IgG and IgA responses. Our results show that HIV-gp140-carrying wax nanoparticles can induce strong cellular/humoral immune responses without inflammation and may be of potential use as effective mucosal adjuvants for HIV vaccine candidates.

## 1 Introduction

The HIV pandemic continues to be a major global health priority, and while there has been good progress in the development of antiretroviral drugs that have contributed to longer survival of infected individuals, prospects of an effective vaccine against HIV remain largely elusive [1,2]. Different strategies to induce effective immune responses to HIV have been attempted in both animal and human models but with little success and controversial results [3,4], although some protective responses have been reported [5,6]. A critical goal of HIV vaccination is the induction of mucosal humoral immune responses. This is predicated on the production of antibodies (Abs) with capacity of hindering the entrance of HIV and its subsequent interaction with target cells at mucosal sites either by viral neutralization, aggregation, or Fc receptor mediated mechanisms [7]. Because HIV antigens (Ags) alone induce very low if any immune responses, the use of adjuvants is of paramount importance. Adjuvants being molecules, compounds or macromolecular complexes that boost the potency and longevity of specific immune responses to Ag with little toxicity and long-lasting immune effects [8]. Biodegradable nanoparticles (NP, <700 nm) have been studied extensively as vehicles for delivery of Ag to antigen presenting cells (APCs) making them good adjuvant candidates [9–14]. NP can enhance the effectiveness of Ag uptake, which then increases Ag delivery to intracellular compartments of APC such as dendritic cells (DCs) and macrophages [15]. Hence, NP may increase Ag presentation capacity, thus boosting cellular and humoral immune responses.

The Ag delivery capacity of NP has been shown both in vitro and in vivo for a wide array of Ags such as tetanus toxoid [16], *Neisseria meningitides* [17], *Bacillus anthracis* [18], and HIV Ags [19–22]. These studies provide evidence that NP may be an important tool for Ag delivery and subsequent induction of cellular and humoral immune responses, critical for development of vaccines. However, success in the development of NP as delivery systems of vaccines has been previously hampered by their low level of colloidal stability and wide limitations in manufacturing scale-up.

We have developed NP made of yellow carnauba (YC) wax with high colloidal stability, low cost and scalable manufacture that would provide a rapid product development pathway. These YC-wax NP can efficiently adsorb Ags such as tetanus toxoid (TT) and the trimeric form of HIV Ag CN54-gp140 (gp140), and are readily internalized by APC with subsequent induction of cellular and humoral immune responses both in vitro and in vivo. In particular, Ag-adsorbed NP enhanced T-cell proliferation responses in human PBMC (TT) and mouse splenocytes (HIV gp140). Also, gp140-adsorbed NP greatly enhanced serum IgG and IgA after systemic immunization and, more importantly, induced high levels of vaginal IgG and IgA after intranasal immunization.

## 2 Materials and methods

### 2.1 Particle production

Solid lipid NP were prepared using a low pressure melt-emulsify-chill (MEC) process. A molten yellow carnauba (YC) wax (Koster Keunen, Watertown, CT) was dispersed into a hot aqueous emulsifier solution under control shear and then cooled to yield a stable dispersion of solid lipid NP. For the preparation of fluorescence NP, the oil-soluble fluorescent dye Pyrromethene-567A (emission wavelength 546 nm, Exciton, Dayton, OH) was encapsulated in the NP. Cationic, anionic and non-ionic emulsifiers comprised of long carbon chains were used to stabilize and also modify the surface charge of the NP.

Particle size was determined by photon correlation spectroscopy using a Brookhaven BI90 Plus (Brookhaven Instruments, Holtsville, NY). The zeta (*Z*) potential (a measure of the surface electrical charge) of the NP and Ags was measured in 1 mM KCl by phase analysis light scattering using a Malvern Zetasizer NanoZS90 (Malvern Instruments, Malvern, UK).

Particle morphology was analyzed by electron microscopy. Serial dilutions of the NP in nanopure water were dispensed in 400 nl drops onto a silicon chip, and left to dry. Samples were kept in the sputtering chamber at 5 × 10^2^ mbar for about 4 h, and then sputter-coated with 15 nM gold. All images were taken at 20 kV, and at various magnifications using a Hitachi S3500N scanning electron microscope.

NP colloidal stability was determined by storing 10% solid NP dispersions in glass vials at 5 °C and 25 °C. Particle size and zeta potential were measured over a 12 month period as described above. For viscosity assessment, NP suspensions were stored in 125 ml plastic bottles for the length of the stability studies and the viscosity measured at different time points using a Brookfield viscometer LVT (Brookfield Engineering Labs, Middleboro, MA). Spindle #4 (low viscosity sample spindle) was placed directly in the sample, and speed setting 6 was used for all measurements.

### 2.2 Antigens

A clade C HIV-1 envelope clone p97CN54 was originally isolated from a Chinese patient [23] and was made available by H. Wolf and R. Wagner, University of Regensburg, Germany. Trimeric gp140 (gp120 plus the external domain (ED) of gp41), designated CN54 gp140, was produced as a recombinant product in CHO cells and manufactured to GMP specification by Polymun Scientific, Vienna, Austria. Bovine serum albumin (BSA) and TT were obtained from Sigma–Aldrich, Ayrshire, UK and Statens Serum Institute, Denmark, Copenhagen, respectively.

### 2.3 Antigen adsorption to nanoparticles

Antigens were adsorbed to NP by means of electrostatic interaction after mixing 750 μg of Ag in 1 ml solution with 56.2 μl of a 1% solids NP solution, and incubated at room temperature for 30 min. To determine binding of Ag to the NP, the *Z* potential of NP was tested before and after protein adsorption, since proteins modify the NP surface charge. Adsorption was also tested by Bradford assay, and for gp140 a specific anti-gp140 ELISA was performed. Because the NP are made of wax material, it was not possible to spin down the NP for further testing of unbound Ag present in the supernatant, therefore a different protocol had to be used. After incubation of NP with Ag, the mix was spun at 4000 × *g* for 10 min using a 1,000,000 MW cut-off Vivaspin filter (Sartorius Stedim Biotech, Goettingen, Germany). Antigen alone was spun in parallel to control for the amount of Ag retained in the filter. After centrifugation, the NP were retained in the filter and the amount of Ag present in the filtrate was then tested by Bradford and ELISA assays. To determine the amount of Ag adsorbed to NP, the amount of Ag detected in the colorimetric assays was calculated as a percentage of the amount of Ag alone recovered after filtration.

Co-adsorption of Ag with the TLR-9 ligand CpGB or PolyI:C was performed using the YC-Brij700-chitosan NP, which are positively charged. CpGB (Eurofins MWG Operon, Ebersberg, Germany) and Poly (I:C) (Invivogen, San Diego, CA) was added to the NP-Ag complex at 4.25 μg/ml final concentration and incubated for an additional 30 min. CpGB and Poly (I:C) binding was assessed using the PicoGreen dsDNA and RiboGreen dsRNA quantitation reagents (Invitrogen Ltd., Paisley, UK).

### 2.4 Isolation and culture of human dendritic cells

Buffy coats obtained from healthy volunteers were used for separation of mononuclear cells (MNC) by density gradient centrifugation using ficoll-hypaque (Histopaque, Sigma). Monocytes were separated from non-adherent cells by adherence to plastic using complete medium (CM: RPMI-1640 plus 10 mM HEPES, 2 mM l-glutamine, 100 IU/ml penicillin, 100 μg/ml streptomycin, all from Sigma) supplemented with 0.5% AB pooled human serum (PHS, Dynal Biotech, Ullernchausseen, Norway). Adherent cells were then cultured for 4 days with 15 ml CM supplemented with 5% PHS that contained 25 ng/ml GM-CSF and 30 ng/ml IL-4 (R&D Systems, Inc., Minneapolis, MN). Complete medium with cytokines was replaced and after 3 days the cells were recovered and tested for cell morphology by optical microscopy, and for DC phenotype by immunofluorescence and flow cytometry.

### 2.5 Optical microscopy

Cells were placed in glass-bottom culture dishes (MatTek, Co., Ashland, MA) in CM plus GM-CSF and IL-4. The microscope and stage were enclosed within a heated (37 °C) chamber (Solent Scientific, UK) and cells were cultured in 5% CO_2_ in air. Images were captured using an Olympus IX71 inverted fluorescence microscope equipped with a Hamamatsu C4742-95 digital camera, using a 20× objective with an additional 1.6× adaptor. Captured images were analyzed using Image Pro Plus software (Media Cybernetics, USA).

### 2.6 Immunofluorescence and flow cytometry

Assessment of DC differentiation/enrichment and modulation of DC phenotype before and after treatment with gp140-adsorbed NP was determined by single cell surface immunostaining using monoclonal Abs against lineage markers and co-stimulatory molecules. Cells were stained with FITC-labeled anti-CD14, -CD3, -CD19, -CD56, and -DC-SIGN; PE-labeled anti-CD11c, -CD40, -CD80, -CD83, -CD86 and CCR7, and PE-Cy5-labeled-HLA-DR mAb. Ten thousand events were acquired in a FACSort Becton-Dickinson cytometer (San Jose, CA), and the samples were analyzed using the CellQuest software version 3.3 (Becton Dickinson, PaloAlto, CA).

### 2.7 Nanoparticle cell internalization and intracellular tracking

Nanoparticle-Ag cell internalization was tested by flow cytometry and confocal microscopy using Pyrromethene-567A-labeled NP. Cells (DC or THP-1 cells) were cultured at 5 × 10^5^/well in a 24-well plate with CM plus 5% PHS. Pyrromethen-567A-labeled Ag-adsorbed NP were added to the cells at a final dilution in CM corresponding to 5 μg/ml gp140 and incubated overnight.

For flow cytometry analysis, the cells were recovered after culture, were washed with PBS, and fixed with 1.5% formaldehyde. Ten thousand events were acquired and analyzed by flow cytometry as described above.

For confocal analysis, DC were resuspended in 50 μl of PBS containing 5.0 μg/ml red fluorescent Alexa Fluor-594 wheat germ agglutinin (WGA, Invitrogen) to stain the cell membrane. Cells were incubated for 10 min at 37 °C, then washed and fixed for 10 min. After fixation, the fixing buffer was completely removed by centrifugation, and the cells counterstained with Vectashield mounting medium (Vector Laboratories, Peterborough, UK) that contained DAPI. Cells were analyzed by confocal microscopy using a LSM 510 laser scanning microscope (Carl Zeiss MicroImaging, Germany).

Tracking of NP-Ag within DC endolysosomes was assessed using a lysosome specific dye on DC cultured on Lab-tek chamber slides (Nalge Nunc International, Naperville, IL) pre-coated with gelatin. Dendritic cells were cultured overnight in CM containing IL-4 and GM-CSF. The CM was replaced with serum-free medium, and gp140-adsorbed NP at 5 μg/ml Ag, final concentration were added to the wells together with 100 μM Lysotracker Red (DND-99, Abs 577 nm; Em 590 nm, Invitrogen) prewarmed at 37 °C in serum-free medium. The cells were incubated for 2 h at 37 °C after which the serum-free medium was replaced with CM, and analyzed by confocal microscopy.

### 2.8 In vitro activation of human DC by antigen-adsorbed nanoparticles

Differentiated immature DC were cultured in the presence of GM-CSF + IL-4, with or without gp140-adsorbed NP (5 μg/ml final Ag concentration). Modulation of DC activation/maturation was tested after 24, 48, and 72 h by determining cell surface expression of CD40, CD54, CD80, CD83, CD86, CCR7, and HLA-class II using immunostaining and flow cytometry, and by assessing cytokine/chemokine release in the cell culture supernatants by multiplex assay. DC cultured in CM only were used as a negative control of stimulation, and in the presence of 25 ng/ml TNF-α as a positive control.

### 2.9 Cell proliferation assays

#### 2.9.1 Human cells

Human MNC (2 × 10^5^ cells/200 μl) were stimulated for 5 days with 12.5 μg/ml TT in CM plus 5% PHS. Because nearly 100% of the TT was adsorbed to the NP (see Section 3.1), an amount of 12.5 μg/ml was used for both NP-adsorbed and free Ag. Free CpGB and Poly (I:C) were used at a final concentration of 4.25 μg/ml, which was the same amount used for co-adsorption with Ag onto NP. Phytohaemaglutinin (PHA, 5 μg/ml, SIGMA) was used as a positive control of stimulation, and CM alone as a negative control. BSA-adsorbed NP, TT plus CpGB without NP, or chitosan alone were also used as controls. Cell proliferation was assessed by incorporation into DNA of [^3^H]Td (GE Healthcare, Buckinghamshire, UK). The cells were pulsed with 0.5 μCi [^3^H]Td/well 18 h before harvesting, and counts per minute (c.p.m.) determined in a liquid scintillation β counter (1450 Microbeta Plus, Wallac Oy, Turku, Finland). Proliferation response was calculated as the mean ± SD of the c.p.m. from three replicates.

#### 2.9.2 Murine cells

Splenocytes from gp140-immune Balb/c mice were cultured for 3 days in the presence of 5 μg/ml gp140, either free or adsorbed to NP. Concanavalin-A (5 μg/ml, Sigma) was used as a positive control of stimulation. After 48 h, the cells were pulsed as for human cells, and 18 h later the cells were harvested and the c.p.m. counted. Proliferation response was expressed as stimulation index (PI), calculated by dividing the mean of the c.p.m. from three replicates of the experimental by the mean c.p.m. of the not-stimulated cells.

### 2.10 ELISA

#### 2.10.1 Detection of antigen-specific antibody responses

Determination of specific TT serum IgG, specific gp140 serum IgG, IgG1, IgG2a, and IgA, as well as specific gp140 IgG and IgA in vaginal and nasal lavages, and in feces was performed by ELISA. ELISA plates (MaxiSorp, Nalge-Nunc International, Rochester, NY) were coated overnight at room temperature with 4 μg/ml TT or 5 μg/ml gp140 in PBS. Blocking was performed for 1 h at 37 °C with PBS containing 1% BSA. Serially diluted samples were incubated for 1 h at 37 °C. Bound IgG, IgG1, and IgG2a were detected by incubation for 1 h at 37 °C with goat anti-mouse Ig-HRP (AbD Serotec, Kidlington, Oxford, UK), or with biotinylated goat anti-mouse IgA Ab (SouthernBiotech, Birmingham, AL) to detect bound IgA. An amplification step was performed to detect IgA by incubating the plates with HRP-streptavidin conjugate (R&D Systems) for 1 h at 37 °C. Plates were developed by adding tetramethylbenzidine (TMB, Pierce-Endogen, Woburn, MA) and incubating the plates in the dark. The reaction was stopped using 1.0 N H_2_SO_4_, and optical densities (O.D.) read at 450 nm. A mix of pre-immune samples was run in 6-replicates per plate and the cut-off calculated (after subtracting the blank) as the mean of these 6 values plus 3 SD, except for that of feces where 5 SD were used.

#### 2.10.2 Detection of gp140

ELISA plates were coated with 1 μg/ml in PBS of affinity purified sheep anti-HIV-1-gp120 polyclonal antibody (AAlto Bio Reagents, Dublin, Ireland) and incubated overnight at room temperature. Blocking was performed for 1 h at 37 °C with Buffer 1 (PBS plus 2% skimmed milk powder). gp140 standards and samples were added to the wells and incubated for 2 h at 37 °C. Detection of gp140 was performed by incubation for 1 h at 37 °C with 2 μg/ml 5F3 anti-gp140 human mAb in Buffer 2 (PBS supplemented with 2% skimmed powder milk, 5% porcine serum and 0.5% Tween-20), followed by incubation for 1 h at 37 °C with goat anti-human IgG-HRP (SouthernBiotech) in Buffer 2. Plates were developed with TMB for 20 min in the dark. The reaction was stopped with 1.0 N H_2_SO_4_ and O.D. read at 450 nm.

### 2.11 Detection of cytokines/chemokines by multiplex assay

Human cytokines/chemokines in cell culture supernatants were detected using an in-house multiplex assay following a protocol recommended by the manufacturers (R&D) as previously described [24].

### 2.12 In vivo immunizations with tetanus toxoid and gp140

#### 2.12.1 Animals

Female Balb/c mice, 6–8 week old, were obtained from Harlan Olac Ltd., UK. Mice were kept at the Biological Research Facility, St. George's University of London. All procedures were performed in accordance with the United Kingdom's Home Office standards under the Animals Scientific Procedures Act, 1986, and approved by the School's Ethical Review Committee.

#### 2.12.2 Immunization protocol and sample collection

**Fig. 1:**
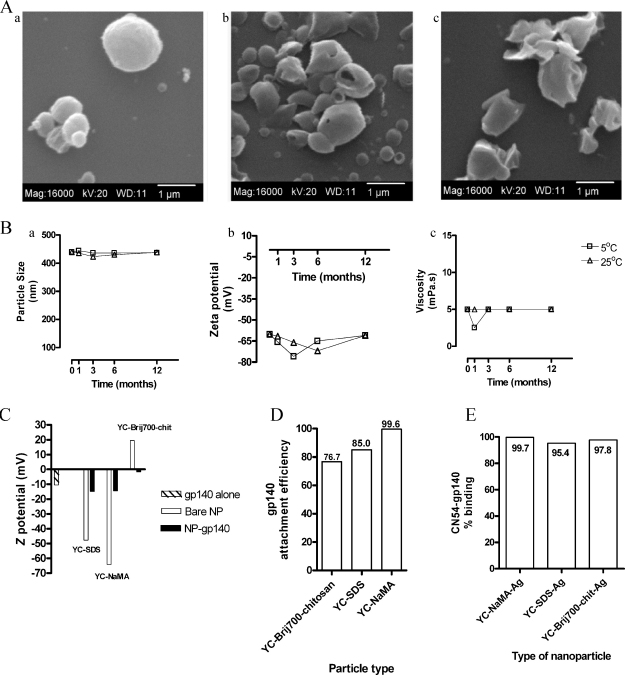
Physicochemical characterization of nanoparticles and binding to antigen. (A) Scanning electron microscopy of YC-wax NP: *a*, YC-SDS; *b*, YC-NaMA; *c*, YC-Brij700chitosan. (B) Colloidal stability of YC-NaMA NP determined up to one year by *a*, particle size distribution (PSD); *b*, zeta potential (Z); and *c*, viscosity. (C) Zeta potential of naked NP and after adsorption with gp140 Ag. (D) Attachment efficiency of gp140 to NP measured by Bradford assay. Numerical values in the figure are the percentage of Ag bound to NP after subtracting the amount detected on the filtrate of Ag alone. (E) Detection by ELISA of gp140 adsorbed to NP. Values inside bars represent the percentage of Ag bound to NP with respect to Ag alone taken as 100%.

Mice were inoculated i.d. with 12.5 μg (TT) or 20 μg (gp140) in a total volume of 100 μl in sterile saline on both dorsal flanks following a prime-boost-boost protocol at 4 (TT) and 3 (gp140) week intervals. For i.n. immunization, 20 μg gp140 with or without NP in a maximum volume of 25 μl were gently dispensed in the animal's nostrils after isofluorane-induced anaesthesia. Antigen-adsorbed NP were prepared the same day of immunization. Fresh components of the formulations were used in these experiments because they were performed in parallel with the NP colloidal stability studies (see Fig. 1B). These studies suggested nonetheless that similar results would be obtained using the same formulation over time. Alum-Ag complex was prepared by mixing equal volumes of Ag and Alum solution (Imject Alum, Pierce, Rockford, IL), and mixed by rotation for 30 min at room temperature.

Blood samples were collected before priming, 1–3 days before boosting, and at 4 (TT) and 3 (gp140) weeks after the last boost. Serum was separated from clotted blood and stored at −80 °C until further use. Vaginal samples were collected by flushing 30 μl of PBS three times into the vagina of anaesthetized animals, pooled and supplemented with 8 μl of a 25× protease inhibitor cocktail (Roche Diagnostics, Manheim, Germany). Samples were incubated for 30 min on ice and then spun at 14,000 rpm for 10 min. Supernatants were collected and stored at −80 °C. Eight fecal pellets/mouse were collected, weighed and mixed with 4× their weight of 1× protease inhibitor cocktail. Samples were homogenized to dissolve the pellets and incubated on ice for 1 h. The samples were spun twice at 14,000 rpm for 10 min, and cleared supernatants stored at −80 °C. Nasal samples were obtained after sacrifice of the animals by flushing the nasal cavity with 300 μl of PBS containing 1× protease inhibitor cocktail. The samples were stored at −80 °C.

### 2.13 Statistical analyses

Analyses were performed using GraphPad Prism, version 4.00 (GraphPad Software). Linear data was expressed as means ± SEM, whereas logarithmic data was expressed as geometric means ± 95% confidence interval. Statistical differences between groups were calculated using one-way ANOVA with Tukey's multiple comparison posttest to compare groups by pairs. Differences between groups in relation to time were analyzed by two-way ANOVA with Bonferroni's posttest for comparison of pairs. Paired Student's *t*-test was used to compare two groups. Differences were considered significant at *P* ≤ 0.05.

## 3 Results

### 3.1 Characterization of YC-wax nanoparticles

Multiple types of YC-NP emulsified with different surfactants were screened for low cell toxicity, efficient cellular uptake, and good protein adsorption (data not shown). Three different YC-NP were selected that met these criteria: YC-SDS (yellow carnauba-sodium dodecil sulphate), YC-NaMA (sodium myristate acetate), and YC-Brij700-chitosan. The latter NP was emulsified with Brij700, a surfactant with a long carbon chain (C18) that contains 100 ethylenoxide (EO) units, and then mixed with medium molecular weight chitosan during the oil-in-water melting process to provide the NP surface with a positive charge.

The zeta potential (*Z*) of the different YC-NP, a measurement in mV of the magnitude of repulsion or attraction between particles, was: YC-SDS, −47.7; YC-NaMA, −64.1; and YC-Brij700chitosan, +19.5. The size of the NP ranged between 387.0 and 675.0 nm, with mean size ± SD for each NP as follows: YC-SDS, 406.5 ± 27.94, *n* = 6; YC-NaMA, 478.8 ± 100.9, *n* = 5; and YC-Brij700-chitosan, 588.0 ± 123.0, *n* = 2. The NP polydispersity index (PDI) was YC-SDS: 0.21 ± 0.033; YC-NaMA: 0.17 ± 0.05; and YC-Brij700chitosan 0.41 ± 0.23. Representative SEM pictures of YC-SDS, YC-NaMA, and YC-Brij700chitosan particles are shown in Fig. 1A. Nanoparticles showed high stability at 5 °C and 25 °C in terms of particle size, ZP, and viscosity for up to 12 months after preparation (Fig. 1B), demonstrating good colloidal stability.

Zeta potential of the Ags, as expected, varied widely depending on the pH due to the amphoteric characteristics of the proteins. However, all three Ags (BSA, TT, and gp140) showed negative ZP at pH ranging between 7 and 8. Interestingly, whereas the ZP at this pH interval was about −10 mV for BSA and gp140, that of TT reached −30 mV. These results suggest that, at physiological pH, adsorption of Ags to the NP may vary depending on both NP and protein surface charge. However, all three Ags bound to anionic and cationic NP (data not shown and Fig. 1C). Binding of gp140 to negatively (YC-SDS and YC-NaMA) and positively (YC-Brij700-chitosan) charged NP is shown in Fig. 1C as indicated by the change in ZP of NP after incubation with gp140. We believe that association of these Ags with the YC-NP may be dominated by both electrostatic and hydrophobic interactions [25].

To further confirm adsorption of gp140 to NP, Bradford assay and gp140-specific ELISA were also performed. Both assays showed that YC wax NP bound gp140 with high efficiency (Fig. 1D and E). Binding of BSA and TT to wax NP, assessed by Bradford, was also highly efficient (data not shown).

### 3.2 YC-wax nanoparticles are internalized by antigen presenting cells

**Fig. 2:**
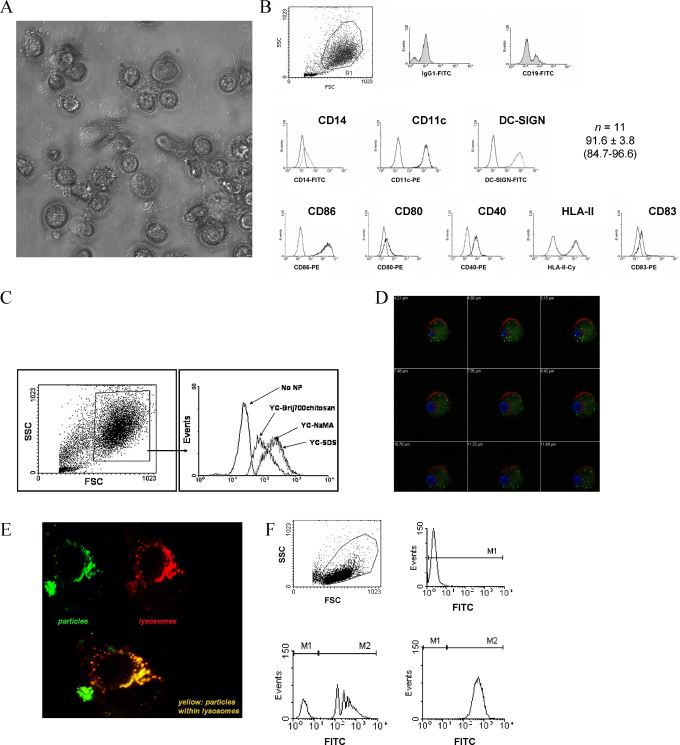
Analysis of gp140-adsorbed nanoparticle internalization by antigen presenting cells. (A) Phase contrast microscopy of DC. (B) Phenotype of in vitro differentiated human DC. Dot plot shows the gated DC population analyzed. Left histograms represent the isotype control. Inset depicts the staining for CD19+ showing about 10% of B cells. *n* represents the number of blood donors with the mean ± SD plus range of cells with a DC phenotype. (C) Flow cytometry analysis of gp140-adsorbed wax NP uptake by DC. Left histograms correspond to cells without NP. (D) DC gp140-wax NP (YC-NaMA shown) uptake as demonstrated by confocal microscopy. Red: cell membrane; Blue: Nucleus; Green: fluorescent gp140-adsorbed YC-NaMA NP. Values at the top left corner of each quadrant represent the distance in μm from top to bottom of the cell after every section. (E) Lysosomal localization of gp140-adsorbed YC-NaMA NP within DC. Yellow color represents the overlapping of green (NP) plus red (lysosomes) colors. (F) Comparison of wax NP uptake by THP-1 cells of two different types of NP: polystyrene (left) vs YC-wax NaMA (right). Top right panel represents the MFI of cells without fluorescent NP.

In vitro human monocyte-derived DC were generated using a standardized protocol as described by Henderson et al. [26] with minor modifications. Blood-derived monocytes were isolated by plastic adherence and showed typical spiky cell membrane projections following 7 days of culture in the presence of GM-CSF and IL-4, as shown in Fig. 2A. Immunostaining and flow cytometry analysis of 11 different DC isolations showed that 91.6% ± 3.8 (range: 84.7–96.6%) of cells had a DC phenotype with very low or negative expression of CD14, and high expression of CD11c, HLA-class II Ags, and DC-SIGN. CD40 and CD86 were consistently highly expressed on these cells, whereas CD80 and the maturation marker CD83 were expressed at low levels (Fig. 2B). The non-DC present in these isolates were consistently B-lymphocytes (Fig. 2B inset). The three YC-wax NP were studied for NP intracellular uptake. Both naked and TT- and gp140-adsorbed YC NP were readily internalized by DC as demonstrated by flow cytometry and confocal microscopy (Fig. 2C and D, respectively). Once internalized, YC NP were localized in endolysosomes (Fig. 2E). Cellular uptake of YC-wax NP was more efficient and was more uniformly distributed within the cell population than that of polystyrene nanobeads (Fig. 2F). Here, 100% of THP-1 cells internalized YC-wax NP whereas about 70–90% of these cells internalized polystyrene NP.

### 3.3 gp140-adsorbed YC-wax NP do not induce activation of DC neither by modulating cell surface activation/maturation markers nor release of cytokine/chemokines

Human monocyte-derived DC were stimulated with gp140-adsorbed YC-wax NP (YC-SDS, YC-NaMA, and YC-Brij700-chitosan) and expression of the cell surface markers CD40, CD54, CD80, CD83, CD86, CCR7, and HLA-class II Ags was assessed by immunofluorescence and flow cytometry after 24, 48, and 72 h post-stimulation. There was no effect on the expression of these molecules, even when tested at an extended time point of 72 h (data not shown). Likewise, there was no cytokine/chemokine induction by YC-wax-gp140-adsorbed NP (data not shown). Naked NP also did not induce any DC activation.

### 3.4 YC-wax-nanoparticles enhance T-cell proliferation responses to antigen in human PBMC and mouse splenocytes

**Fig. 3:**
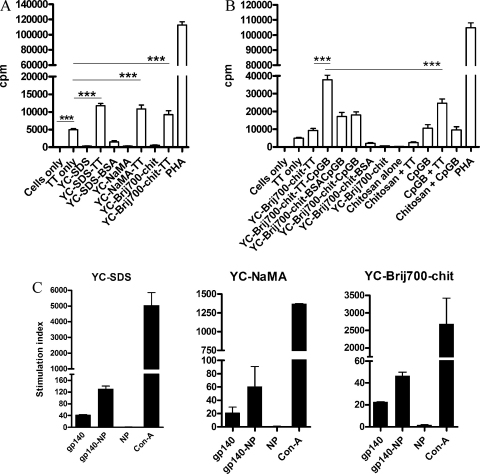
Enhancement of T-cell proliferation responses to antigen after adsorption to nanoparticles. Human T-cell proliferation response to (A) TT-adsorbed NP, and (B) TT after co-adsorption to YC-Brij700-chitosan NP with CpGB. TT was used at 12.5 μg/ml and CpGB at 4.25 μg/ml. Results shown are the mean ± SEM of c.p.m. values of three different donors performed in triplicate cultures. ***P < 0.001. (C) Mouse splenocyte proliferation response to gp140-adsorbed NP. Splenocytes from gp-140 immunized mice were stimulated in vitro with gp140-adsorbed NP at 5 μg/ml final Ag concentration. Results shown are the mean ± SEM of the SI of two different experiments performed in triplicate. Mean comparisons were performed by one-way ANOVA.

We sought to determine whether YC-wax NP would enhance the T-cell proliferation responses to Ag. Since there are some limitations for the use of gp140 to induce human T-cell proliferation in vitro such as the lack of immune response in HIV unexposed healthy volunteers, and the anergic status of many HIV-infected individuals, TT was used as a model Ag. Hence, we tested the capacity of TT-adsorbed YC-wax NP to enhance T-cell proliferation in fresh PBMC from healthy volunteers. As shown in Fig. 3A, YC-wax NP enhanced T-cell proliferation to TT. This response was independent of the type of particles since both negatively (YC-wax SDS and YC-wax NaMA) and positively (YC-wax Brij700-chitosan) charged NP enhanced T-cell proliferation responses to TT (*P* < 0.0001). This response was specific for TT as YC-wax NP adsorbed with the irrelevant Ag BSA did not induce T-cell proliferation (Fig. 3A). Interestingly, when the TLR-9 ligand CpGB (Fig. 3B) but not the TLR-3 ligand Poly I:C (data not shown) was co-adsorbed with TT to YC-Brij700-chitosan NP, the T-cell proliferation response was further enhanced (*P* < 0.0001). To confirm that this effect was due to the co-adsorption of both TT Ag and CpGB to the YC-wax NP, several controls were performed (Fig. 3B). Specifically, to test that the enhancing effect was not due to cell activation induced by the chitosan present on the YC-wax Brij700-chitosan NP, both chitosan alone and together with TT (in the absence of NP) were also assessed. Results show that neither chitosan nor TT+chitosan enhanced T-cell proliferation (Fig. 3B). In addition, although CpGB induced T-cell proliferation on its own, this induction was significantly lower than that induced by TT-CpGB co-adsorbed NP. Further confirmation of the enhancing effect on T-cell proliferation by co-adsorption of TT plus CpGB on NP, was demonstrated when instead of using TT, the irrelevant Ag BSA was co-adsorbed to NP with CpGB (Fig. 3B).

To test whether NP could enhance T-cell proliferative responses to gp-140, splenocytes from gp140-immunized mice were used in vitro. Splenocytes were cultured in the presence of Ag alone or gp140-adsorbed NP and the incorporation of ^3^H[Td] into DNA measured after three days of culture. gp140-adsorbed NP but not naked NP enhanced splenocyte proliferative responses to gp140 (*P* < 0.001)(Fig. 3C), indicating that such an effect was not due to the particles themselves.

### 3.5 YC-wax NP enhance systemic humoral immune responses to antigen in vivo

**Fig. 4:**
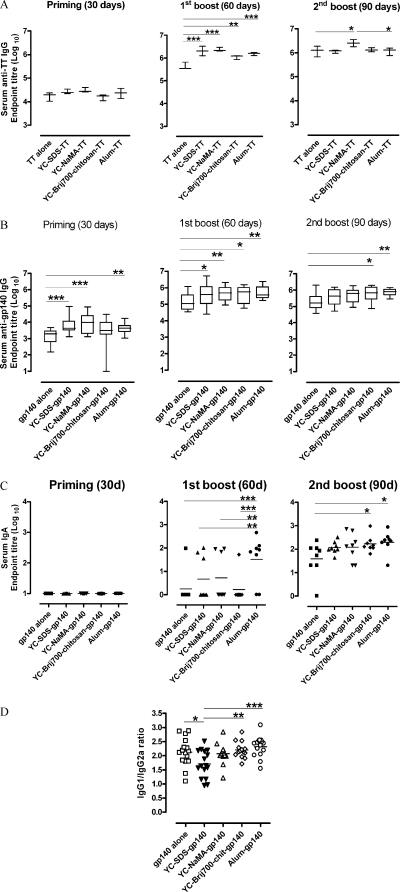
In vivo humoral immune responses to antigen-adsorbed nanoparticles. Kinetics of specific serum IgG after i.d. immunization with (A) TT and (B) gp140. Groups of 3 (TT) and 8 (gp140) mice were immunized three times with (A) 12.5 μg TT and (B) 20 μg gp140 either free or adsorbed to NP. Ag adsorbed to Alum was used as a positive control of immunization. Serum samples were obtained before each immunization and 30 days after the last immunization, and tested for IgG end-point titers by ELISA. Results shown are the mean ± SEM of one experiment (TT) and two aggregated experiments (gp140). Mean differences were analyzed by two-way ANOVA with Bonferroni posttest to compare means by pairs. *P < 0.05; **P < 0.01; ***P < 0.001. (C) Kinetics of specific serum IgA after i.d. immunization with gp140. Groups of 8 mice were immunized and sampled as in (B), and the serum samples were tested for specific-gp140 IgA end-point titers by ELISA. Mean comparisons were analyzed as in (B). (D) gp140-specific serum IgG1/IgG2a ratio. Serum samples obtained 30 days after the third immunization were tested by ELISA for gp140-specific IgG1 and IgG2a end-point titers. Plotted are the aggregated data of IgG1/IgG2a Log_10_ ratio values of two different experiments for the groups shown in the figure. Means were compared by one-way ANOVA. *P < 0.05; **P < 0.01; ***P < 0.001.

Experiments were performed in mice using gp140-adsorbed NP to determine whether NP can enhance humoral responses to Ag in vivo. Similar experiments were performed previously using TT and results showed that systemic immunization with all three NP enhanced serum levels of specific anti-TT IgG after the first boost (60 days), which were comparable to those induced by Alum (Fig. 4A). Such levels were not enhanced further after the third immunization (90 days), and became comparable to those induced by TT alone, which by itself is a very potent Ag [27], suggesting that the role of NP was to increase the kinetics of serum anti-TT IgG. For induction of specific anti-gp140 IgG and IgA, animals were immunized i.d. with gp140 following a prime-boost-boost protocol at 30 day intervals. Serum samples were taken before each immunization and 30 days after the last boost, and the levels of IgG and IgA were tested by gp140-specific ELISA. gp140 alone induced significant levels of IgG but these levels were much higher when the Ag was adsorbed to NP (Fig. 4B). Such IgG levels were comparable to those induced by Alum (day 60), and differences were already observable following a single prime (day 30). Plateau IgG levels were already observed after first boost (day 60, Fig. 4B).

Serum specific anti-gp140 IgA was only detected after the second immunization (60 days) but the titers were not high because the maximum end-point titer levels of IgA detected under these immunization conditions were 282.4 ± 88.4 (log_10_ 2.45 ± 1.95). YC-Brij700chitosan-gp140 but not YC-SDS-gp140 nor YC-NaMA-gp140 promoted significant specific-gp140 IgA titers (*P* < 0.05) after three immunizations (90 days). Such effect was comparable to that of Alum at the same time point (*P* < 0.05). However, the effect of NP as a whole on serum specific-gp140 IgA after i.d. immunization was low because the kinetics and magnitude of specific-gp140 IgA responses promoted by Alum after the first boost (60 days) was significantly superior to those of NP (Fig. 4C).

To test whether YC-wax NP modulated T-helper cell responses, the gp140 specific IgG1/IgG2a ratio was also determined by ELISA. Of note, gp140 alone induced an IgG response that was biased towards a Th2 phenotype. Such a response did not appear to be modulated by Alum, YC-wax NaMA or YC-wax Brij700-chitosan (Fig. 4D). However, YC-wax SDS appeared to induce a more balanced Th1/Th2 response (Fig. 4D).

### 3.6 YC-wax-nanoparticles enhance mucosal humoral responses to gp140

To test whether NP were also capable of enhancing mucosal humoral responses to gp140, mice were immunized nasally with either Ag alone or adsorbed to YC-wax-NaMA NP, and the levels of IgG and IgA were determined in serum and mucosal fluids. We chose YC-NaMA NP for i.n. immunization first because, these NP showed a significant enhancement of systemic humoral immune responses to both TT and gp140 across the i.d. immunizations (see Fig. 4A and B). Second, NaMA is a naturally occurring surfactant, present in many natural oils and, more importantly, in human nasal fluid [28]. Alum was not used as a positive control of adjuvanticity for i.n immunization due to the intrinsic inflammatory role of Alum salts, since part of their mechanism of action is to induce necrotic and damaged cells at the site of injection [29], an effect that would be incompatible with nasal immunization.

**Fig. 5:**
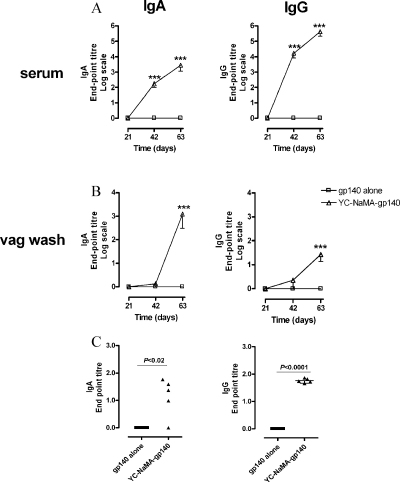
Systemic and mucosal humoral immune responses to gp140-adsorbed nanoparticles after intranasal immunization. (A) and (B) Kinetics of serum and vaginal IgG and IgA levels. Groups of 5 animals were immunized i.n. with 20 μg of gp140 either free or adsorbed to YC-NaMA NP. Serum and vaginal lavages were obtained before each immunization and 21 days after the last immunization, and tested for specific-gp140 IgG and IgA end-point titers by ELISA. Means were compared by two-way ANOVA. ***P < 0.001. (C) Levels of IgG and IgA in nasal lavage after second boost. Animals were immunized as in (A) and (B), and 21 days after the last immunization the animals were sacrificed and the nasal cavity washed with saline. Samples were tested for gp140-specific IgG and IgA end-point titers as for (A) and (B). Means were compared by paired *t*-test.

Antigen alone failed to induce any response (Fig. 5). In contrast, there was a steady increase over time in both serum IgG and IgA in response to gp140 adsorbed to YC-NaMA NP (Fig. 5A). These levels did not seem to reach a plateau after the second boost, as it was observed with serum IgG after intradermal immunization. Notably, high levels of IgA were also observed in vaginal secretions, with a moderate increase in IgG (Fig. 5B). In addition, IgG and IgA levels were also detected in the nasal lavages of these mice (Fig. 5C). No antibody induction was observed in feces (data not shown). Of note, the IgG1/IgG2a ratio in serum was very close to 1 (1.57 ± 0.079), which was lower than that induced by intradermal immunization with gp-140-YC-wax NaMA, suggesting that the type of T-helper immune response induced by NP may change depending on the route of immunization.

## 4 Discussion

We have developed a highly stable NP vaccine delivery system made of YC-wax material. These NP have a low cost of production that is easily scalable. Indeed, similar particles are already manufactured in kilogram quantities for use in the cosmetics industry. These features, together with their capacity to efficiently adsorb protein Ags, to be readily internalized by APC, and to enhance immune responses to Ag both in vitro and in vivo, make them good potential delivery systems for vaccines, and in particular that of HIV vaccines for the developing world.

Manipulation of the YC-wax NP surface charge with surfactants, provides optimal flexibility to adsorb different types of Ag [30]. In this study, Ags as diverse as TT, BSA, and HIV-1 gp140 were efficiently adsorbed to both negatively and positively charged NP. In addition, the surface charge flexibility also facilitated co-adsorption of more than one molecule onto the NP surface as shown by co-adsorption of Ag with CpGB and PolyI:C. After screening a large range of wax NP, three different types were selected according to their low toxicity, Ag adsorption efficiency, and cell internalization profile, i.e., YC-SDS, YC-NaMA, and YC-Brij700-chitosan. The first two NP had a net negative charge, whereas the third one was highly positive, a characteristic defined by the presence of the carbohydrate chitosan. We determined adsorption of gp140 to these NP by three different methods: *Z* potential, Bradford assay, and ELISA. All three methods provided strong evidence of effective Ag adsorption to NP. In addition, the ELISA assay suggested that antigenicity was unaffected, which may represent an advantage over Ag encapsulation as reported previously for a form of HIV-gp120 by Singh et al. [31].

Flow cytometry and confocal microscopy studies clearly showed that Ag-adsorbed YC NP were readily internalized by APC, and that these NP were subsequently tracked within endolysosomes, suggesting that the NP may have the capacity to deliver Ag into the Ag processing and presentation compartment.

Naked YC-wax NP did not induce cytokine/chemokine production or up-regulation of co-stimulatory molecules on DC in vitro, nor induced visible signs of inflammation after both mucosal and systemic administration in vivo (data not shown). This lack of DC activation by naked NP is important especially if used at the urogenital tract, because such cell activation would induce mucosal inflammation at this level that may facilitate HIV infection.

Antigen-adsorbed YC-wax NP (TT in human PBMC and gp140 in mouse splenocytes) enhanced T-cell proliferation responses in vitro. The response to TT by human PBMC was greatly enhanced by co-adsorption with CpGB (Fig. 3B) but not with PolyI:C (data not shown). CpGB on its own enhanced cellular proliferation, and we speculate that CpGB induces non-specific proliferation of PBMC most likely due to polyclonal B cell activation, as has been described previously [32]. Nevertheless, the enhanced proliferation observed with co-adsorption of TT + CpGB particles was significantly greater than the additive effect of TT plus CpGB alone.

Nanoparticles enhanced serum humoral immune responses to Ag in vivo. Animals immunized i.d. with gp140-adsorbed NP enhanced serum IgG production after a single prime, and this effect was comparable or better than that induced by Alum. Surprisingly, CpGB co-adsorbed to NP with either TT or gp140 did not enhance antibody production further (data not shown). Alum salts are well known strong parenteral adjuvants which are components of an array of licensed human vaccines [8]. However, to date they have not been successfully used for mucosal vaccination. Reactogenicity of Alum salts is an important characteristic of their adjuvanticity. Their mechanism of action has been associated with induction of local uric acid crystals [33] and inflammasome activation with release of IL-1β by macrophages and DC [34,35]. Such reactogenicity is deemed too potent for mucosal use [36]. We do not know the mechanism of in vivo enhancement of humoral responses by gp140-adsorbed NP but since NP alone showed little if any reactogenicity in the skin of mice when compared to that induced by Alum, the mechanism of action may be highly different to that of Alum salts. The efficient cell internalization of NP and their subsequent localization within the endolysosome compartment in the absence of co-stimulatory molecule up-regulation and cytokine/chemokine production by DC clearly suggest a different mechanism. Thus, the lack of Alum-type reactogenicity of NP makes them good potential candidates for mucosal immunization. This may be particularly important where potential inflammation and edema have been associated with induction of Bell's palsy [37]. Although no adverse effects were observed on nasal administration of YC-NaMA NP in mice, further experiments will be required to confirm the safety of these NP after intranasal application in humans, in particular the assessment of the effect of surfactants on the nasal olfactory and respiratory epithelia. Nevertheless, the amount of NaMA, a naturally occurring fatty acid in human nasal fluid [28], used in this formulation was very low (0.025%), and as such the likelihood for toxicity is considered to be small.

We immunized mice with gp140-adsorbed YC-NaMA using different routes of immunization, including nasal, vaginal and rectal. The responses to gp140 via vaginal and rectal mucosal compartments were weak or null (data not shown). Reasons for this unresponsiveness in these mucosas may include physical properties of mucus (pore size and rheological factors) [38], and/or their paucity of follicle associated epithelium when compared to nasal associated lymphoid tissue (NALT). Nasal immunization, in contrast, potently induced both systemic and mucosal humoral immune responses. Intranasal immunization has been described as an effective route to induce systemic and mucosal immune responses to Ag, in particular in the urogenital tract, with scarce if any induction in the gut [39,40]. When mice were immunized nasally with gp140-adsorbed NaMA NP, high levels of IgG and IgA were induced in serum as well as vagina, and titers were still increasing following three immunizations. IgA levels in serum induced by i.n. immunization were around one to two orders of magnitude higher than those induced by i.d. immunization, suggesting that the NP themselves do not inherently drive IgA switching. We believe it is more likely that the route of immunization has an important role at inducing serum IgA as has been previously suggested [39,40]. We speculate that gp140-specific IgA plasma cells induced in the nasal cavity may home to spleen or bone marrow. It is worth noting that levels of gp140-specific IgG and IgA were also enhanced in the nasal cavity. This suggests that wax NP may also have utility for delivering of immunogens against respiratory pathogens. M-cells of NALT are thought to play an important role in the uptake of NP in rodents and humans and are absent in vaginal and rectal mucosa [41–43].

The nasal route has been extensively studied not only for vaccination purposes [44–47] but also for the delivery of drugs [48], and NP have been used nasally to induce immune responses to TT [49] and HIV [50]. Induction of systemic and mucosal immune responses to HIV after nasal immunization of mice [51,52], guinea pigs [51] and macaques [5] with HIV-gp120 Ag has been described previously. In the latter, serum and vaginal Ab responses were induced after nasal immunization only when followed by one or two intramuscular boosts. These levels were highly enhanced in vagina after challenge with SHIV, suggesting that the nasal priming induced effective memory responses at mucosal level [5]. In our mouse model, three nasal immunizations were enough to induce high levels of IgG and IgA in serum and vagina. It remains to be confirmed whether this immunization protocol with NP will work similarly in macaques or humans, or whether these Abs would be neutralizing. Therefore, further studies are warranted that assess homologous and heterologous immunization protocols to determine the feasibility of using these NP, as effective delivery systems of HIV Ags, in the development of mucosal vaccination in humans.

## Disclosure

Particle Science Inc has IP rights and economical interests in carnauba wax based nanoparticles mentioned in this article.
